# Use of High-Frequency In-Home Monitoring Data May Reduce Sample Sizes Needed in Clinical Trials

**DOI:** 10.1371/journal.pone.0138095

**Published:** 2015-09-17

**Authors:** Hiroko H. Dodge, Jian Zhu, Nora C. Mattek, Daniel Austin, Judith Kornfeld, Jeffrey A. Kaye

**Affiliations:** 1 Department of Neurology, Layton Aging and Alzheimer’s Disease Center, Oregon Health & Science University, Portland, Oregon, United States of America; 2 Oregon Center for Aging and Technology (ORCATECH), Oregon Health & Science University, Portland, Oregon, United States of America; 3 Department of Neurology, Michigan Alzheimer’s Disease Center, University of Michigan, Ann Arbor, Michigan, United States of America; 4 Graduate School of Public Health, University of Michigan, Ann Arbor, Michigan, United States of America; 5 Portland Veterans Medical Center, Portland, Oregon, United States of America; University of Glasgow, UNITED KINGDOM

## Abstract

**Background:**

Trials in Alzheimer’s disease are increasingly focusing on prevention in asymptomatic individuals. This poses a challenge in examining treatment effects since currently available approaches are often unable to detect cognitive and functional changes among asymptomatic individuals. Resultant small effect sizes require large sample sizes using biomarkers or secondary measures for randomized controlled trials (RCTs). Better assessment approaches and outcomes capable of capturing subtle changes during asymptomatic disease stages are needed.

**Objective:**

We aimed to develop a new approach to track changes in functional outcomes by using individual-specific distributions (as opposed to group-norms) of unobtrusive continuously monitored in-home data. Our objective was to compare sample sizes required to achieve sufficient power to detect prevention trial effects in trajectories of outcomes in two scenarios: (1) annually assessed neuropsychological test scores (a conventional approach), and (2) the likelihood of having subject-specific low performance thresholds, both modeled as a function of time.

**Methods:**

One hundred nineteen cognitively intact subjects were enrolled and followed over 3 years in the Intelligent Systems for Assessing Aging Change (ISAAC) study. Using the difference in empirically identified time slopes between those who remained cognitively intact during follow-up (normal control, NC) and those who transitioned to mild cognitive impairment (MCI), we estimated comparative sample sizes required to achieve up to 80% statistical power over a range of effect sizes for detecting reductions in the difference in time slopes between NC and MCI incidence before transition.

**Results:**

Sample size estimates indicated approximately 2000 subjects with a follow-up duration of 4 years would be needed to achieve a 30% effect size when the outcome is an annually assessed memory test score. When the outcome is likelihood of low walking speed defined using the individual-specific distributions of walking speed collected at baseline, 262 subjects are required. Similarly for computer use, 26 subjects are required.

**Conclusions:**

Individual-specific thresholds of low functional performance based on high-frequency in-home monitoring data distinguish trajectories of MCI from NC and could substantially reduce sample sizes needed in dementia prevention RCTs.

## Introduction

As clinical trials progress from safety to efficacy phases the cost of development increases dramatically [1, 2]. This is related to a number of factors, not the least of which is the large sample size that may be needed to show a potential effect [1–4]. The need for large samples is often driven by the inaccuracy of estimating changes because the outcome measures have high variability, not only due to measurement errors, but also due to inherent fluctuations in individuals’ abilities to perform certain tasks. These measurement errors and individual fluctuations could be offset by highly frequent assessments which lead to more accurate or precise longitudinal trajectory estimates of outcomes [3, 5]. However, in most clinical trials, only a sparse number of measurements, such as every year or every six months, are available. Although there have been significant advances in early phase drug development to feed the pipeline of testable compounds, there has been little progress in changing the paradigm for improving the conduct of trials so as to speed the time needed to obtain an answer as to efficacy or to reduce the number of subjects needed to find that answer. In this paper we propose a new approach to improving the conduct of clinical trials that combines the capability of acquiring much more frequent and objective data using ubiquitous home-based sensing and computing methodologies for data capture. The approach generates high frequency data which can provide person-specific distributions of outcomes within a short duration of follow-up. The distributions can then be used to capture person-specific changes or shifts over time. We show that this approach can provide adequate statistical power with reduced sample size requirements.

To demonstrate the potential value of this new approach, we use the example of designing a treatment trial for the prevention of Alzheimer’s disease (AD), an area of great unmet need for effective therapies. The need for prevention trials for Alzheimer’s disease is highlighted by the fact that recent experimental drug trials in established AD have failed leading to the view that in order for treatment to be effective, earlier presymptomatic intervention is needed [6, 7]. Thus, trials in AD are increasingly focusing on secondary prevention in asymptomatic individuals. For example, among recently launched large clinical trials for AD are several targeted to prevent further CNS amyloid beta protein (Aβ) accumulation in vivo during a pre-symptomatic stage [8] or among those destined to have Aβ aggregation and subsequent dementia by virtue of carrying autosomal dominant genetic mutations in the presenilin 1 gene related to amyloid processing [9, 10]. The duration from the time when Aβ begins to accumulate until AD symptoms appear is now estimated at about 15 years or more [11], providing an ample window of opportunity for prevention. However, during the pre-symptomatic phase, cognitive function and functional abilities are not often detected as declining using sparsely-obtained conventional clinical assessment approaches. This poses a challenge in examining treatment effects among pre-symptomatic participants [3, 4, 12, 13].

In the current study, we used Oregon Center for Aging and Technology (ORCATECH) in-home continuous assessment approach, where activity- and health-related metrics are created from round-the-clock data collected by an unobtrusive in-home sensor system (http://www.orcatech.org). The approach provides sufficient data points to generate individual-specific distributions of functional outcomes, such as computer usage and walking speed and their variability within a short time period (e.g., 3 months). These in-home activity data have been shown to differ in trajectories of change among MCI as compared to age-matched controls [14–16]. Our objective was to compare sample sizes required to achieve sufficient power to detect prevention trial effects in two scenarios: (1) annually assessed neuropsychological test scores modeled as a function of time using mixed effects models (a conventional approach), and (2) likelihood of subject-specific low performance modeled as a function of time using mixed effects models. We first obtained the empirical effect size, which is the difference in trajectories (time slopes in outcomes) between those who remained cognitively normal and those who developed MCI during an average of 3 years of follow-up by using the two types of outcomes above (annually assessed neuropsychological test scores and likelihood of individual-specific low performance). Using the difference in empirically identified time slopes between those remaining normal during the follow-up (normal control, NC) and those who developed MCI, we estimated sample sizes required to achieve 80% statistical power for detecting 20% 30% or 40% treatment effects. (i.e., the difference in time slopes between NC and MCI would be reduced by 20%, 30% or 40%, respectively).

## Materials and Methods

### Data

The data comes from a longitudinal cohort study, Intelligent Systems for Assessing Aging Change (ISAAC). Participants were recruited from the Portland, Oregon, metropolitan area through advertisement and presentations at local retirement communities. Details of the study protocol for ISAAC have been published elsewhere [14]. Briefly, entry criteria for the study included being age 70 or older, living independently (living with a companion or spouse was allowed, but not as caregiver), not demented (Mini-Mental State Examination [17] ≥ 24; Clinical Dementia Rating (CDR) [18] scale score ≤ 0.5), and in average health for age. Medical illnesses that would limit physical participation (e.g., wheelchair bound) or likely lead to untimely death (such as certain cancers) were exclusions. A total of 265 participants were enrolled beginning in 2007. The participants lived in a variety of settings—from apartments in organized retirement communities to freestanding single-family homes. One hundred nineteen participants living alone were included in the current analysis.

### In-home activity data

The ISAAC research protocol and the in-home monitored activities collected in the study have been described previously [14]. For the current paper, we selected the following three person-specific in-home activity variables shown to be correlated with cognitive function in our previous studies: Weekly mean walking speed, weekly walking speed variability and weekly home computer usage. Briefly, daily in-home walking speed was calculated using a line of four motion sensors positioned in a series on the ceiling. The field of view of the sensors was restricted so they fired only when the participant passed directly underneath them. The distance between sensors was recorded to allow adequate calculation of velocity as the participant passed through the line of sensors. Data from sensors were received by a dedicated research laptop computer placed in the participant’s home, time-stamped and stored in an SQL database. All data were automatically uploaded daily to a central database in the project data center. A detailed description of the algorithm and its validation process are found elsewhere [19, 20]. Weekly walking speed variability was generated by calculating the Coefficient of Variation (COV: the ratio of the weekly standard deviation to its mean multiplied by 100 (a dimensionless number)) [15]. Weekly average daily home computer usage was measured as follows. Computer sessions were calculated using mouse movement data. Each mouse movement of more than five pixels generated a Windows event that was saved and time stamped. Each day was partitioned into 5-minute periods, and for any period with more than 100 mouse events, the computer was considered in use. The total time on the computer per day was then estimated as the sum of these 5-minute in-use periods, measured in minutes. Mean daily use (in minutes) was the sum of total time on the computer per week divided by total number of days with use in the week. A more detailed description of the computer use metric is found elsewhere [16]. We previously found that although average time spent on computer per day was not different between groups at baseline, there was a significant decline in usage over time among those with MCI as compared to cognitively intact participants [16].

These in-home activities are collected unobtrusively (no wearable technology was used) and continuously, i.e., the data are generated 24/7. This data was used to examine the differences in slope of these variables over time between those with intact cognition and those who transited to MCI (incidence MCI cases) defined as CDR = 0.5 during the annual in-home clinical exam described below and used for power calculations.

### Annual clinical examination and neuropsychological tests

In addition to continuously obtained in-home activities, participants were also assessed clinically at baseline and during annual visits in their home using a standardized battery of prevailing clinical tests consisting of physical and neurological examinations. MCI incidence cases defined as CDR [18] = 0.5 in the current study was confirmed during this annual exam. The annual neuropsychological test results over time were used to generate empirical data to see the difference in longitudinal trajectories (slope differences) between those with intact cognition and those who transited to MCI, and used for power calculations. Neuropsychological tests considered to be representative of 5 cognitive domains were administered: Logical Memory Immediate and Delayed Recall (memory) [21], Category Fluency (executive function) [22], the Trail Making Test Part A (psychomotor speed) and B (executive function) [23], the Wechsler Adult Intelligence Scale-Digit Symbol (attention) [24], and the Boston Naming Test (language) [25]. CDR [18] was determined independently from the neuropsychological test results.

### Ethics Statement

Study protocol and consent forms were approved by the Oregon Health & Science University Institutional Review Board. All participants provided written informed consent.

## Statistical Analysis

### Empirical Data

#### Annually assessed neuropsychological tests as outcomes (conventional approach)

We first examined time slope differences on annual neuropsychological tests using mixed effects models between those who developed MCI (defined as the incidence of CDR = 0.5 with at least one subsequent assessment being CDR = 0.5) and those who remained cognitively intact during the follow-up. Among the MCI incident subjects, the data points prior to transition were included, while those after transition were excluded because our aim here is to estimate the difference in slopes during the pre-symptomatic period (before MCI designation). We estimated the difference in slope between the two groups using a group-by-time interaction term (with MCI as the reference group). The coefficient of the interaction variable shows how much less decline the normal group experienced over time as compared with incident MCI subjects.

#### In-home monitoring derived activities as outcomes

**Fig 1:**
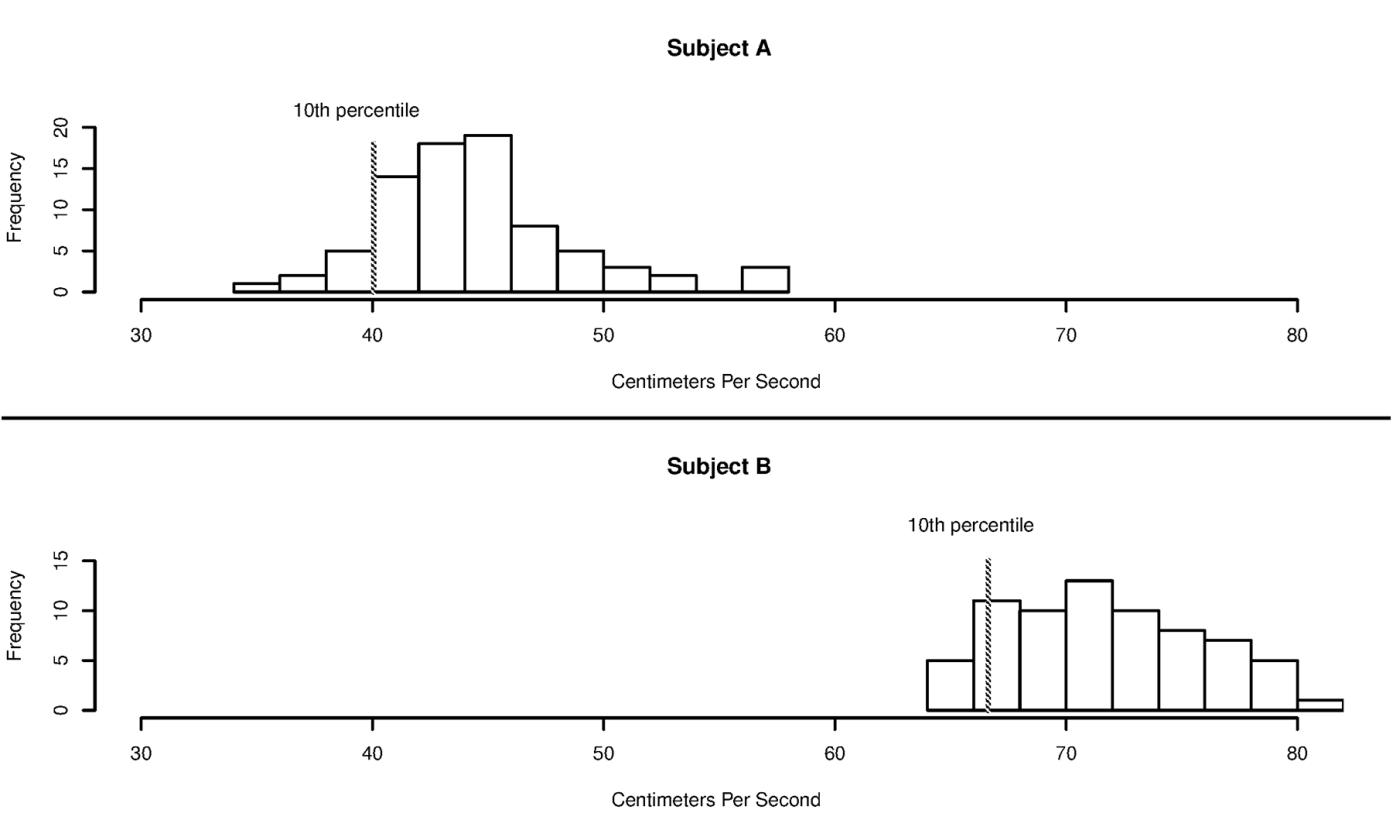
Examples of subject-specific distributions of walking speed. NOTE: According to the baseline (first 90 days) walking speed histograms, subject A (id = 7621) was much slower initially than subject B (id = 11012). However, subject A was only slower than his/her subject specific baseline 10th percentile during 11% of the later weekly follow-ups, and subject B was slower than his/her subject specific baseline 10th percentile during 79% of the weekly follow-ups. This indicates that although subject A was slower at the beginning, his/her walking speed was stable while there was an obvious slowing trend for subject B. The group’s 10th percentile based on the first three months of data is 39.3. Subject B was never slower than the group 10^th^ percentile threshold during the entire follow up period. Therefore the fact that subject B got much slower over time was not reflected by using the group specific threshold.

As with the annually assessed neuropsychological tests, we first fit mixed effects models examining the slope difference between cognitively intact and incident MCI groups. The data points observed after MCI incidence were not included. Second, we calculated each participant’s distributions of weekly mean walking speed, weekly walking speed variability and weekly computer usage (time in minutes spent on their home PC) using the data observed during the first 90 days (approximately 3 months). This data allowed us to generate individual-specific distributions of each activity and several measures of their variability such as mean, median, 1 standard deviation (SD) below mean, 1SD above mean, 10^th^ percentile person-specific low threshold, etc. For example, Fig 1 shows walking speed data generated within the first 3 months of data accumulation from 2 different individuals. Using these person-specific distributions, we fit generalized linear mixed models with outcomes being likelihood of experiencing values below the “person-specific” lowest 10^th^ percentile, 20^th^ percentile, 30^th^ percentile, 40^th^ percentile and 50^th^ percentile thresholds (for walking speed and computer usage) and the values above the “person-specific” highest 70^th^ percentile, 80^th^ percentile and 90^th^ percentile thresholds (for walking speed variability). We used this approach because our prior studies suggested that variability of in-home monitored activities might increase before subjects transitioned to MCI [15]. That is, mean values could be relatively stable over time even though variability in functional outcomes increases for each subject. Linear mixed effects models, where trajectories of marginal mean values over time are estimated, ignore the likelihood of subjects experiencing extremely low (or high) outcome values. We calculated sample sizes required to achieve 80% statistical power using the results of the linear mixed effects models (differences in mean trajectories of neuropsychological tests between the two groups) and the results of generalized mixed models (differences in likelihood of low performance on in-home monitored activities defined using baseline person-specific distributions of outcomes).

### Sample size estimates

The percentage effect size is the proportion of reduced decline out of the expected maximum amount of decline. For example, those who developed MCI had an annual decline of 1 unit in a measure of our interest, while those who remained normal had 0.3 unit decline per year, the latter considered as age-associated normative decline. The difference is 0.7 units which is the expected maximum amount of reduction in decline attainable for any treatment since treatment is conservatively assumed not to improve outcomes beyond age-associated normal decline. If the percentage effect size is 30%, then the treatment group will have a reduction in annual decline by 0.21 unit (0.7 X 0.30 = 0.21), that is, 0.79 (1–0.21 = 0.79) unit decline per year, while the placebo group would have an annual decline of 1 unit. For mixed effects models, sample size is calculated by using a well-established formula [26]. The sample sizes required to achieve 80% power for the generalized linear model were estimated using Monte Carlo simulations: A fitted generalized linear mixed model with adjusted empirical effects (for example 50% effect size) was used to simulate 1000 replicates of data of the given sample size (assuming equal size for both treatment and MCI groups) and specified time points in days (for example, four years of data had time ranges from 0 to 1456 days (4yrs x 52weeks x 7days). For each replicate of simulated data, the same generalized linear mixed model was applied, and we reject the null hypothesis if the estimated effect size (group difference on slope) is significantly different from 0 with significance level = 0.05. Lastly, we estimated the power by calculating the rejection rate over 1000 replicates. We assumed that drop-out rates would not be different between methods so this was not included in the models.

## Results

### Baseline characteristics

Table 1 shows the baseline characteristics of subjects included in this study. Among 119 subjects, 17 subjects developed MCI (CDR = 0.5) during the average follow-up of 3.8 years. Among 17 incident MCI cases, no one returned to CDR = 0 in subsequent assessments during the follow-up period. Those who developed MCI had lower scores on Logical Memory Immediate Recall (p = 0.008) and Delayed Recall scores (p = 0.004) at baseline, but no other differences were found.

**Table 1:** Baseline characteristics (means or percentages given with SD in parentheses).

	Total (N = 119)	Normal Controls (N = 102)	MCI Incidence (N = 17)	p-value
Age (years)	84.42(5.07)	84.16(4.86)	86.04(6.16)	0.09
% Male	15.11%	14.17%	21.05%	0.73
Years of Education	15.41(2.33)	15.53(2.38)	14.63(1.86)	0.09
Duration of Follow-up in years	3.80(1.17)	3.80(1.21)	3.84(0.93)	0.88
Duration of Follow-up in years before MCI incidence			2.24(1.33)	N/A
Number of annual clinical assessments (for the MCI group, the assessment numbers before MCI incidence)	4.15(1.36)	4.41(1.19)	2.53(1.26)	N/A
**In-Home Continuously Monitored Data** ^**#**^				
Mean Walking Speed (cm/sec)	62.59(17.83)	63.02(18.19)	60.03(15.71)	0.48
Mean Daily Computer Usage (minutes)^##^	78.11(57.34) (n = 97)	73.91(51.81) (n = 86)	110.94(86.08) (n = 11)	0.19
**Neuropsychological Assessments**				
Category Fluency (animals and vegetables)	30.98(7.19)	31.31(7.17)	28.89(7.18)	0.19
Trail Making Test A (time in seconds)	42.66(18.86)	41.80(17.10)	48.11(27.52)	0.34
Trail Making Test B (time in seconds)	124.81(60.37)	120.81(57.45)	151.50(73.46)	0.10
Digit Symbol Test	39.42(9.71)	40.08(9.18)	35.32(12.06)	0.11
Logical Memory Immediate Recall	13.40(3.93)	13.67(4.05)	11.74(2.54)	0.008**
Logical Memory Delayed Recall	12.05(4.05)	12.37(4.13)	10.05(2.84)	0.004**
Boston Naming (30 items)	25.86(3.22)	25.93(3.24)	25.42(3.15)	0.52

**: p<0.01.

#: Baseline week

##: PC usage was not assessed for all subjects.

### Empirical results

**Fig 2:**
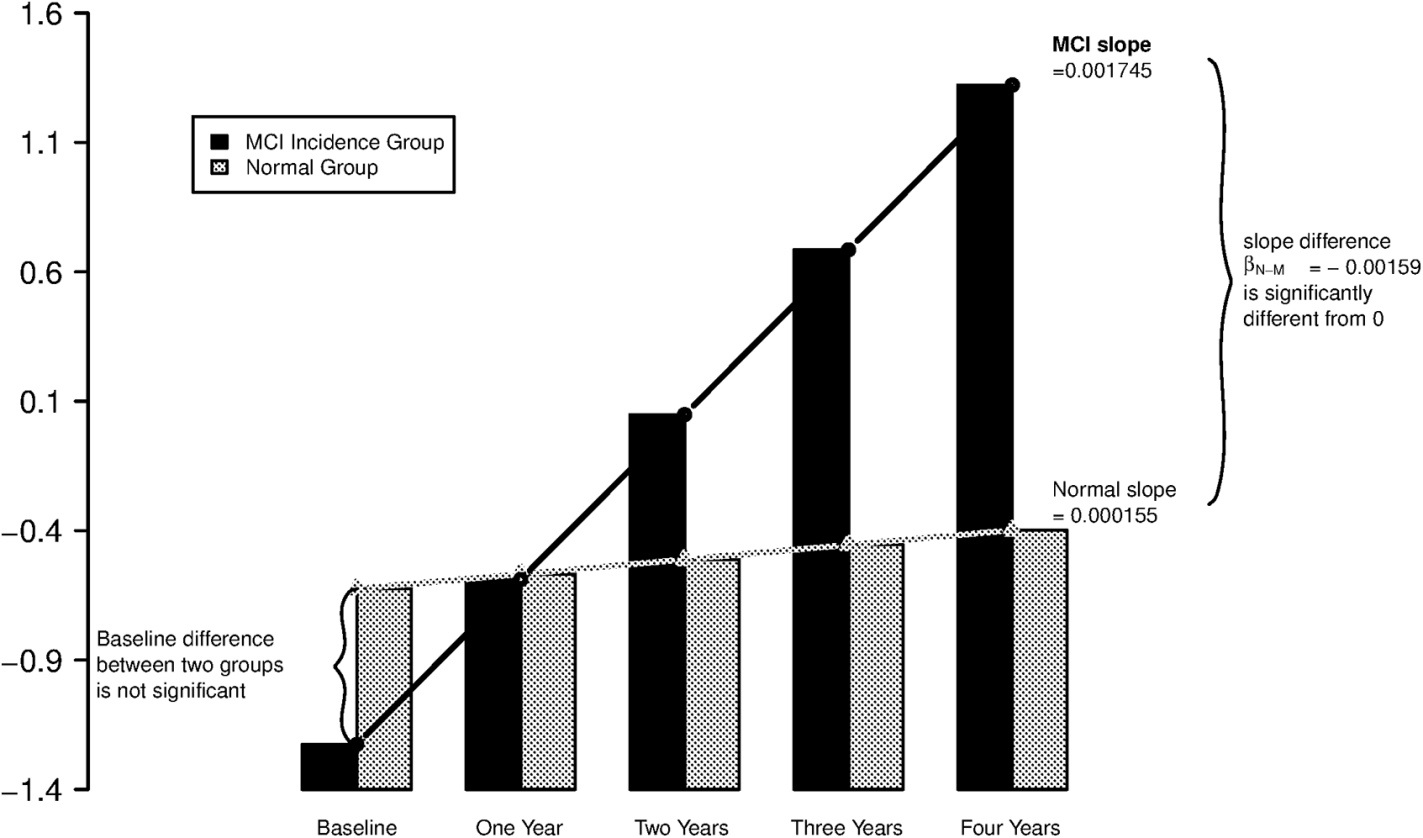
Likelihood (log odds) of days with low threshold computer usage over time. Example: Computer use. For each participant, we calculated the 40th percentile of the first available 90 days of daily records of computer usage level (in minutes) and defined his/her individual-specific 40^th^ percentile low threshold. Weekly average data based on these 90 days of daily records were then excluded from analysis, and the first week after these 90 days was defined as the baseline week of computer usage for this participant in our analysis. Model description detail is provided in Supplemental Material.

As shown in Table 2 (column A), no neuropsychological tests demonstrated a significant difference in trajectories between groups over the observation period. As for in-home activities, only computer usage demonstrated a significant difference in trajectories between the two groups (p = 0.01). The time scale in these models is in number of days. Normal subjects (those who maintained normal cognition) had less decline over time in weekly average minutes on computer than incident MCI subjects (i.e., positive direction in coefficient). For example, at the end of one year, normal subjects spent 29% more time in computer usage (exp(7 days X 52 weeks X 0.0007)) compared with those who transitioned to MCI. For generalized mixed effects models, we examined the likelihood of subjects experiencing functional outcomes from 10^th^ percentile- to 50^th^ percentile- below individual specific thresholds by 10% increments (i.e., 10, 20, 30, 40 and 50) and reported the most and the second most significant results in Table 3 (column) for each activity measure. As expected from our previous studies, those destined to develop MCI spent fewer minutes on their computer over time [16] and their walking variability increased [15]. The table can be read as follows. For example, the likelihood of weekly average time spent on a personal computer falling below the subject-specific 40^th^ percentile threshold is significantly less among normal subjects; on average, compared with the incident MCI group; the odds of a normal subject experiencing this threshold within a day is 99.8% (exp(-0.0016) = 0.998, that is, about 0.2% lower, or 44.1% (1- exp(-0.0016 X 52 weeks X 7 days)) less at the end of one year (Fig 2). Likewise, the likelihood of weekly walking speed variability falling higher than the subject-specific 70^th^ percentile threshold is about 27.9% less after one year among the normal group compared with the MCI incident group. Likelihood of walking speed falling below subject-specific low thresholds was not significantly different between normal and MCI incident groups.

**Table 2:** Expected outcomes and total sample size estimates: conventional approach using annual neuropsychological test results.

		Empirically Derived Slope Differences			Clinical Trial Sample Size Estimation (estimates based on 4 years of follow-up)			
Model	Outcome	Difference in slope (MCI group as a reference)*	Standard error	p-value	Treatment effect size 20% **	Treatment effect size 30%	Treatment effect size 40%	Treatment effect size 50%
	Category Fluency (animal+vegetable)	0.0026	0.0033	0.43	8050	3578	2013	1288
	Trail Making Test A	-0.0074	0.0086	0.39	6800	3022	1700	1088
	Trail Making Test B	-0.0211	0.0265	0.43	7500	3334	1876	1200
Linear mixed effects model	Digit Symbol	0.0012	0.0046	0.8	75900	33734	18976	12144
	Logical Memory Immediate Recall	0.0017	0.0017	0.32	4900	2178	1226	784
	Logical Memory Delayed Recall	0.0019	0.0017	0.28	4300	1912	1076	688
	Boston Naming (30 items)	0.0006	0.0014	0.66	26800	11912	6700	4288

* Empirical effect size: group effect on slope/slope for normal group (i.e., MCI groups as a reference group). Trajectory after onset of MCI is not included in the slope estimation (i.e., only trajectory up to onset was used). Equal allocation to placebo and treatment groups is assumed.

** Effect size: when MCI incidence group is the reference group, the normal (control) group can be treated as a group with an 'improved' effect. In clinical trial we assume that the treatment group would have 20%-50% of the improvement defined by the effect of normal group.

**Table 3:** Expected outcomes and total (placebo and treatment group combined) sample size estimates: continuous activity monitoring approach using cutoff thresholds derived from individual specific distributions of daily activities observed during the 1^st^ 3 months of in-home monitoring data.

		A. Empirically Derived Slope Differences			B. Clinical Trial Sample Size Estimation (estimates based on 4 years of follow-up)			
Model	Outcome	group effect on slope (Normal vs Incidence)*	standard error	p-value	Treatment effect size 20%**	Treatment effect size 30%	Treatment effect size 40%	Treatment effect size 50%
	walking speed	0.0038	0.0115	0.74	92600	41156	23150	14816
Linear Mixed Effects Models	computer usage***	0.0007	0.0003	0.01	1100	490	276	176
	walking speed variability	0.0021	0.0022	0.34	7550	3356	1888	1208
	walking speed: likelihood of 10^th^percentile low	-0.0008	0.0005	0.1	588	262	148	94
	walking speed: likelihood of 50^th^percentile low	-0.0001	0.0002	0.65	14550	6468	3638	2328
Generalized Linear Mixed Effects Models	computer usage: likelihood of 30^th^percentile low	-0.0014	0.0002	<.0001	76	34	20	14
(with Random Intercept)	computer usage: likelihood of 40^th^percentile low	-0.0016	0.0002	<.0001	58	26	16	10
	walking speed variability: likelihood of 70^th^percentile high	-0.0009	0.0003	0.0009	184	82	46	30
	walking speed variability: likelihood of 80^th^percentile high	-0.0009	0.0002	0.0001	192	86	48	32

* Empirical effect size: group effect on slope/slope for normal group (i.e., MCI groups as a reference group). Trajectory after onset of MCI is not included in the slope estimation (i.e., only trajectory up to onset was used). Equal allocation to placebo and treatment groups is assumed.

** Effect size: when MCI incidence group is the reference group, the normal (control) group can be treated as a group with an 'improved' effect. In clinical trial we assume that the treatment group would have 20%-50% of the improvement defined by the effect of normal group.

***: Log transformed weekly mean PC usage (mean daily PC usage in minutes per week). Equal allocation to placebo and treatment groups is assumed.

### Sample sizes needed to achieve 80% power

Using the empirical results above (i.e., difference in slopes observed between MCI and normal groups), we estimated the sample size required to achieve 80% power to detect a difference between placebo and treatment groups assuming a follow-up period of up to 4 years. Column B in Tables 2 and 3 show the required sample size to achieve 80% statistical power (with α = 0.05) for different desired effect sizes. Since none of the annually assessed neuropsychological tests were significantly different in decline over time between the two groups, these tests as outcomes require large sample sizes to obtain sufficient effect sizes. For a 30% effect size, annually assessed neuropsychological tests require at least 1900 subjects (using delayed recall as the primary outcome). As for in-home activities, if the likelihood of having lower computer usage per day was modeled as a primary trial outcome, a 30% effect size could be obtained with 26–34 subjects; for walking speed variability, 82–86 subjects would be needed.

## Discussion

Clinical trials which shorten the time needed to prove efficacy or reach study endpoints with reduced sample sizes are of significant public health importance because these features not only save trial costs, but also accelerate the translational process from discovery of potential treatments to the availability of treatments for patients [1, 2, 5, 7]. In the current study, we proposed a new approach for improving the conduct of clinical trials using high frequency and objective data derived from in-home monitoring of everyday activities. The system allowed us to capture individual-specific distributions of various in-home activities generated within a short baseline interval. Using individual-specific thresholds for low or high levels of activities and their variability derived from individual-specific distributions, we could substantially reduce estimated sample sizes required to obtain adequate statistical power to show desired effects. For this simulation, we examined two activities obtained through in-home monitoring that have been tied to development of MCI, mobility change (walking speed) and computer use (time on computer). A number of other everyday activity measures could also be examined continuously such as adherence to taking daily medications using an electronic pill box [27], socialization (e.g., time out of home [28, 29], phone usage [30], conversational interactions or speech characteristics [31]), or sleep measures (e.g., time in bed, wake after sleep onset [32]), These all have the intrinsic advantage of not being surrogate markers, but relevant ecologically valid outcomes in their own right [30].

### High-frequency in-home monitored data for RCT

In an ideal secondary prevention AD clinical trial, the goal would be to recruit individuals at risk of developing MCI (and subsequently AD) and to show that the treatment prevents or delays the clear onset of MCI. However, it may take a decade to complete such a study with this endpoint. Thus, in most trials, surrogate outcomes (e.g., results of validated neuropsychological tests or composite scores) are used and the trial is aimed to detect a reduction in their rate of decline (change) in cognitive and/or functional outcomes among the treatment group relative to the placebo group. The US Food and Drug Administration currently outlines this approach for treatments targeted toward pre-symptomatic or incident AD [33]. In principle, one can reduce the number of individuals to be followed or the time of follow-up needed, if one can (1) more precisely estimate the true trajectory of change (increase precision) and (2) use outcomes that detect subtle changes in underlying pathological processes. High-frequency in-home monitoring data could reduce sample size needs because the data facilitates an increase in precision as well as the ability to capture changes effectively by using person-specific distributions, instead of applying group norms or group averages to estimate change. Modeling the likelihood that activity measures fall below a given threshold level (or in the case of variability, go above a threshold) using generalized mixed effects models turned out to be very effective in assessing changes because it takes into account the increase in variability in activity measures, not just mean values in the measures. The approach is especially advantageous in capturing changes which occur during the early pre-symptomatic stage of the dementing disease since previous work suggests that variability could increase during the transition from normal cognition to MCI [15].

Sample size estimates can vary depending on the signal-to-noise ratio and variance and covariance structures derived from the empirical data used to estimate the sample size. As shown in the results from empirical data, computer usage tended to show more significant differences between the incident MCI and normal groups, because this outcome has a higher signal-to-noise ratio as compared to the other outcomes. Although walking speed and its variability have been shown to be associated with cognitive function in various studies [15, 34–37], they can also be affected by non-cognitive comorbidities. The smaller sample size required for computer usage may due to this activity being less affected by potential physical comorbidities in general.

Given that a clinical trial would be targeted to reduce cognitive decline leading to MCI among those with intact cognition at baseline, sample size power is generally estimated using empirically derived trajectories (slopes) among the cognitively intact subjects (subjects who did not develop MCI or AD at least during the study follow up, i.e., non-pathological or normal aging) and among subjects who developed MCI during the follow-up, with the group difference providing an upper bound on potential treatment effects. Using simulated data, Leoutsakos et. al., [12] examined how much trial power could be improved by increasing the sample fraction that would develop AD in the absence of intervention (i.e., increasing the fraction of *true at-risk subjects* at recruitment). In the study, they found that if a biomarker is used with a positive predictive value of 0.5 within 2 years (i.e., half of those at risk subjects enriched by the biomarker will develop AD in the absence of intervention within 2 years) then the power is about 0.71, but if a biomarker is used with a positive predictive value of 0.8 (80% of at risk subjects are those who will transit to AD in the absence of intervention within 2 years), then the power will increase to 0.95 with 200 subjects per arm (Table 2, in [12]). Donohue et al., [13] used empirical data to estimate the minimal difference detectable given 80% power with 30% attrition and a 5% α level, using Aβ positivity to enrich at-risk subjects. Their estimate showed that with 500 subjects per group (placebo and treatment groups), the minimal detectable difference in declines in ADCS-PACC scores (composite scores of episodic memory, timed executive function and global cognition) is smaller than the difference empirically observed in the Australian Imaging, Biomarkers, and Lifestyle Flagship Study of Ageing (AIBL) and ADCS studies, confirming the feasibility of the proposed A4 study (the Anti-Amyloid Treatment in Asymptomatic Alzheimer’s study [8]) with Aβ positivity as an enrichment strategy and ADCS-PACC as the primary outcome [13]. However, this approach, requiring biomarker enrichment means hundreds or thousands of patients need to be screened to enroll the sample needed for the trial. As discussed in the recent summary article by Dorsey et al., [5], technologies used in the current and other studies could play an important role in cost-effective enrichment of clinical trial study participants in the future. Proof of concept clinical trials are required to confirm the idea of using high frequent monitored data as a study enrichment strategy.

### Annual Assessment of Neuropsychological Tests and Sample Size Estimates

In the current study, we showed sample sizes needed using annually assessed neuropsychological tests for a comparison with sample sizes needed using high-frequent in-home monitored data. Our estimate showed larger required sample sizes for annually assessed neuropsychological tests. The current results on the estimated sample size using annually assessed neuropsychological tests are in line with another study where the empirical data is derived among pre-symptomatic subjects in the Alzheimer’s Disease Neuroimaging Initiative (ADNI) data set. Grill [4] estimated required sample sizes per arm for a 36 month trial to detect differences in changes in cognitive and functional outcomes using ADNI baseline biomarker information. Among those with normal cognition, even if the sample is enriched with ApoE e4 carrier status, about 2300 subjects are required using the Clinical Dementia Rating scale (CDR) sum of boxes as an outcome, and 27,380, 8146 and 1237 subjects are required when the primary outcomes are psychometric test scores, the ADAS-Cog, MMSE, and RAVLT-delayed recall, respectively. As the authors noted, the relatively large estimated sample size is due to the fact that the control group in the ADNI I study is heterogeneous in terms of the risk of becoming MCI or AD in the future. If targeted study participants are MCI instead, and with ADAS-Cog as an outcome, the required sample size to achieve a 25% effect size is reported to vary from 375 to 9500+, depending on the assumptions used in the power calculations [3]. More recent analyses [38] showed approximately 1200 subjects (568 per arm) are required to achieve a 20% effect size, given cerebrospinal fluid Aβ1–42 concentration positivity-enriched MCI participants with semiannual outcome assessments for 2 years.

### Cost

The cost of the technologies used in the home monitoring system can be considered small relative to potential benefits and compared to other methods being used to aid detection of change. This is especially the case for pre-symptomatic or early MCI where biomarkers are used in part because subtle clinical or functional changes are difficult to capture with sparsely spaced in person visits. The sensors and hardware used are composed of off-the-shelf components; total costs are in the US$1200-$2000 range. By comparison assessment methods currently used to track pre-symptomatic change such as biomarker studies may cost this amount or considerably more. For example, PET imaging in some markets costs $5000 in the United States for a single scan depending on the study and the ligand. Biomarkers are not necessarily predictive of trajectories of clinical outcomes with infrequent follow-up intervals [39] and could further add noise to outcomes [40]. On the other hand, once placed in the home, the home sensing system remains on for many months or years providing ecologically valid data on a continuous basis that directly speaks to meaningful function (e.g. mobility, computer use, medication adherence). The calculations presented in this paper estimate that required sample sizes may be reduced, for example, ten-fold or more, potentially leading to a large reduction in trial costs.

### Privacy Concerns

The home based assessment approaches used in this new methodology need to be mindful of potential privacy concerns. However, the research platform presented here has been guided by the principle that technologies should not be overly obtrusive or threatening to an individual’s sense of privacy or security, so that we can use the system widely in the community. Although systems are installed to monitor activity, they are unobtrusive and do not record any pictures or uniquely identifying features of the subject. Interception of data during broadband transmission is prevented by data encryption and unauthorized access to the central server is prevented by firewall protection and password-restricted access.

### Study Limitations

Study limitations include: results may be affected by subject enrollment characteristics. As noted, the cohort consisted of highly educated older adults. We did not screen for amyloid risk (genetic mutations, CSF or imaging biomarkers). In this sense they are more likely to represent the general population of patients. The subjects who developed MCI had greater memory impairment on cognitive testing at baseline which is a MCI profile that has been more commonly associated with AD [41]. We derived prevention effects empirically using observed trajectories of MCI incidence cases and those who maintained normal cognition. Actual prevention effects change depending on the base proportion of at-risk subjects [12]. Ultimately the results presented here must be empirically tested in a proof of concept randomized controlled trial design. Given the lack of effective AD treatments and the large number of potential compounds that could be tested, expeditious application of these and other novel trial methods is critically needed so that we may more efficiently and cost-effectively identify efficacy signals for critical “go—no go” decisions in AD treatment programs. Finally the approaches (methods and outcomes) proposed here are not limited to AD, but can be extended to other treatment trials such as treatment studies of pain or mobility disorders.

## Conclusions

High-frequency in-home monitoring data can provide individual-specific thresholds of critical functional performance from data accumulated within a short period of time. Using this approach may effectively reduce needed sample sizes for prevention RCTs. Additionally the monitored activities are ecologically valid outcomes in their own right. Future studies applying this method to various trial outcomes are warranted to validate the generalizability of this approach in clinical trials.

## Supporting Information

S1 Text Example: Statistical Models for Computer Usage Analyses. (DOCX)

S2 Text Codebook for Data. (XLSX)

S1 Data Data for annually assessed neuropsychological tests over time. (SAS7BDAT)

S2 Data Data for weekly computer usage over time. (SAS7BDAT)

S3 Data Data for weekly walking speed over time. (SAS7BDAT)

S4 Data Data for weekly walking speed variability over time. (SAS7BDAT)
