# Supplementary material for: Use of High-Frequency In-Home Monitoring Data May Reduce Sample Sizes Needed in Clinical Trials
Source: PLoS One. 2015 Sep 17;10(9):e0138095. doi: 10.1371/journal.pone.0138095 (PMC4574479; doi:10.1371/journal.pone.0138095)
Supplement: S1 Text — (DOCX) [file pone.0138095.s001.docx]

**Supporting information**

**S1. Example: Statistical Models for Computer Usage Analyses**

For each participant, we calculated the 40th percentile of the first available 90 days of daily records of computer usage level (in minutes) and defined it as his/her specific 40th low threshold. Weekly average data based on these 90 days of daily records were then excluded from analysis, and the first week after these 90 days was defined as the baseline week of computer usage for this participant in our analysis models illustrated as bellows.

Let $L_{it}$ denote whether the $i$th participant's computer usage level hits his/her specific 40th low threshold at time $t$ (in days, for instance baseline week refers to $t=0$, week 1 refers to $t=7$, and so on), and also define $N_{i}=1$ if this participant belongs to the normal group, and $N_{i}=0$ otherwise, then the generalized linear mixed effect model for $L_{it}$ is

$$\mathrm{logit}\left( \Pr\left( L_{it}=1 \right) \right)=\alpha_{M}+\alpha_{N-M}*N_{i}+\alpha_{0i}+\beta_{M}*t+\beta_{N-M}*N_{i}*t,$$

where$\alpha_{0i}\sim N\left( 0,\sigma_{0}^{2} \right)$ denotes individual subject variation apart from the group average at baseline. In addition, $\alpha_{M}$ denotes the average baseline log odds for the MCI Incidence group, $\alpha_{N-M}$ denotes the baseline difference between normal and MCI incidence groups on average,$\beta_{M}$ denotes the daily rate of change in log odds for the MCI incidence group, and $\beta_{N-M}$ denotes the difference in daily rate of change between normal and MCI incidence groups.

The model is estimated using SAS Proc GLIMMIX procedure. The estimated slopes are $\hat{\beta}_{M}=0.001745$ for the MCI incidence group and $\hat{\beta}_{N}=\hat{\beta}_{M}+\hat{\beta}_{N-M}=0.001745-0.00159=0.000155$ for the normal group. The slope difference between the two groups $\hat{\beta}_{N-M}$ is significantly different from 0 with a p-value<0.0001.

The baseline odds are then

$\Pr\left( L_{i0}=1 \right)/(1+\Pr\left( L_{i0}=1 \right))$ = ${exp(\alpha}_{M}+\alpha_{N-M}*N_{i}+\alpha_{0i}))$.

At time $t$, the odds are $\mathrm{odds}_{M}\left( t \right)=exp(\alpha_{M}+\beta_{M}*t)$ if using the MCI incidence group average, and are $\mathrm{odds}_{N}\left( t \right)={exp(\alpha}_{M}+\alpha_{N-M}+\beta_{M}*t+\beta_{N-M}*t)$ for the normal group. Therefore, with one year increase in time, the odds for the MCI incidence group are proportionally increased to

${\mathrm{odds}_{M}\left( t+365 \right)=exp(\beta}_{M}*365)*\mathrm{odds}_{M}\left( t \right)=189\%{*odds}_{M}\left( t \right)$.

Similarly the odds ratio for the normal group between one year later and any starting time $t$ is 106%:

$${\mathrm{odds}_{N}\left( t+365 \right)=exp[(\beta}_{M}+\beta_{N-M})*365]*\mathrm{odds}_{N}\left( t \right)=106\%*\mathrm{odds}_{N}\left( t \right).$$

This indicates that the odds for the normal group are increasing at a significantly lower rate with the difference between the odds ratios being

${{exp[(\beta}_{M}+\beta_{N-M})*365]}/{{exp(\beta}_{M}*365)}$=${exp(\beta}_{N-M}*365)$=${106\%}/{189\%}$= 0.56.

Sample size estimation procedure:

We first estimate the parameters (intercept, slope, within subject variance for the random intercept, group effect on the intercept and group effect on the slope) from the GLMM based on the observed data. After setting the significance level to p=0.05, we start with an initial sample size, and simulate 1000 replicates of data, which consists of years of weekly outcomes from the GLMM with the estimated parameters. We then calculate the power. If the calculated power is less than 80% we then increase the sample size, and if the calculated power is greater than 80% we then decrease the sample size. The new sample size is then used to simulate another 1000 replicates of data, and the procedure is repeated until we find the sample size that yields 80% of power.
